# Longevity

**Published:** 1894-04

**Authors:** 


LONGEVITY; with a list of persons known to have lived one hundred years or over. A reprint from *Virginia Medical Monthly*, February, 1894. By Archer E. Atkinson, M. D., of Baltimore, Md., late Professor Practice of Medicine, ex-member of the Maryland Academy of Sciences; ex-member of Medico-Chirurgical Society of Maryland, and member of the Baltimore Microscopic Society; Late Resident Physician at Greenbrier, White Sulphur Springs, W. Va.

This is a curiosity in its way. The paper contains the names of more than 250 persons who have reached the one hundredth year of and many who have lived far beyond that period. It should be read by medical men and by students of Natural History and of Biology and by statisticians. To be had of the writer. Price, 25 cents a copy.

